# Ethnic Diversity, Ideological Climates, and Intergroup Relations: A Person × Context Approach

**DOI:** 10.5334/pb.465

**Published:** 2019-01-23

**Authors:** Jasper Van Assche

**Affiliations:** 1 Department of Developmental, Personality and Social Psychology, Ghent University, BE

**Keywords:** ethnic diversity, ideological climates, intergroup relations

## Abstract

Intergroup relations represent one of the most difficult and complex knots which we are confronted with in contemporary society. Given that intergroup dynamics permeate all spheres of our daily social lives, it seems vital to systematically investigate how to best predict and explain when, why, and for whom intergroup relations symbolize conflict rather than harmony. This critical review evaluates how the societal context in which individuals live shapes how their personal social-ideological views, their values, norms and beliefs, are associated with their intergroup and related attitudes. Such an approach not only examines psychological/individual and sociological/contextual levels of analysis simultaneously, it also assesses how both work together (i.e., interact) in influencing intergroup relations across various domains of life. In sum, I found that adopting a person × context interaction approach yields interesting and more profound insights in individuals’ attitudes towards ethnicity-, gender, and age-based outgroups, their specifisc expressions of ethnic prejudice (e.g., outgroup negativity, outgroup threat, intergroup contact, and trust within and between ethnic-cultural groups), and even their political attitudes, political party support, neighborhood attitudes and moving intentions.

“Intergroup relations represent in their enormous scope one of the most difficult and complex knots of problems which we confront in our times.”

*Henri Tajfel, 1982*

The topic of intergroup relations is all around us; it seems to be an inevitable and ubiquitous part of social life nowadays. Indeed, themes such as the refugee influx, the Muslim veil, racial profiling, and terrorist threats repeatedly cover the news headlines. Politicians and the public engage in heated arguments on these issues, and opinions are shared in every newspaper we open, on every television channel we watch, and on every social media platform we log onto. It seems that the present multicultural society, consisting of various ethnic and cultural groups, literally divides its citizens’ attitudes and ideas, leaving some to perceive an immanent threat where others see an opportunity to get to know other cultures and habits. The aim of the current review is to add nuance to this polarized debate by scientifically examining the antecedents and consequences of intergroup dynamics in various domains of life within contemporary communities.

Renowned social psychologist Gordon Allport is credited for summarizing the earliest and perhaps most comprehensive body of research on intergroup relations. In his book *The Nature of Prejudice* (1954), he provided a foundation for the study of intergroup dynamics. Since that time, a great deal of work in social sciences has helped to improve our understanding of intergroup relationships. The classic definition of intergroup relations was originally provided by Muzafer Sherif (1966), who suggested that “whenever individuals belonging to one group interact, collectively or individually, with another group or its members in terms of their group identification, we have an instance of intergroup behavior” (p. 12). Hence, intergroup relations (i.e., relationships between different groups of people) can comprise a large range of social groups (e.g., gender-, age-, language-, or sexual orientation-based groups), the most prominent and most-studied being ethnicity-based relations between ethnic-cultural minority and majority members.

Henri Tajfel, a survivor of a World War II prisoner-of-war camp, further highlighted that the potential problem for intergroup relations lies in the overarching natural tendency of human beings to make social categorizations (Tajfel & Turner, 1979; Tajfel 1982). Because of its complexity, individuals are inclined to divide the social world into separate categories. According to Social Identity Theory (Tajfel & Turner, 1979), this process of designating people as a member of one’s own group (i.e., the ingroup; “*us*”) or as member of another group (i.e., the outgroup; “*them*”) occurs almost automatically, and identification with one’s ingroup is almost unavoidable.

Attitudes towards such outgroups can range along a broad spectrum from blatant pronouncements of bigoted sentiment in the form of dehumanization and prejudice, to broad-minded openness, acceptance, and tolerance (Pettigrew, 1969). Whether one’s stances towards intergroup relations are negative or positive depends on an array of factors, and insights from the fields of social psychology, personality psychology, political sciences, and sociology all add a piece to this complex and multifaceted attitudinal puzzle. In that respect, the claim of Tajfel (1982) that intergroup relations represent a “Gordian knot” might still be true thirty-odd years later….

## Intergroup Relations: Conflict or Harmony?

As some of the most pressing, complex, and compelling intergroup phenomena in contemporary Western societies, diversity, multiculturalism, immigration, and integration have become central themes of social science research. Rooted in larger questions concerning the origins of prejudice, stereotypes, discrimination and exclusion, the question of how to predict and explain ethnic majority members’ attitudes, feelings, and behaviors towards minorities is of key relevance to social psychologists.

Psychology has often addressed this question from an individual differences framework, investigating the role of personality traits (e.g., Hodson, Hogg, & MacInnis, 2009; Sibley & Duckitt, 2008), cognitive styles and motivated cognition (e.g., Jost, Glaser, Kruglanski, & Sulloway, 2003; Roets & Van Hiel, 2011), and social-ideological attitudes (e.g., Duckitt & Sibley, 2007; Whitley, 1999; Van Assche, Koç, & Roets, 2019). This last category of individual differences has proven to be among the most robust and reliable predictors of prejudice and outgroup attitudes (see Duckitt & Sibley, 2009). Within his Dual Process Model, John Duckitt (2001) put forward that individuals seem to differ in their propensity to adopt prejudiced and ethnocentric attitudes, and these differences originate from their more general views, beliefs or ideas about how a society should be organized and should function.

Indeed, individuals anchor their outgroup stances in their social-ideological values and beliefs, and these social-ideological attitudes can be divided into two broad and interrelated dimensions. In the social-cultural domain, individuals differ in the value they attach to conformity to traditional norms and values, social cohesion, and collective security (versus openness to experience and individual autonomy). This dimension is often labelled *Right-Wing Authoritarianism* (RWA; Altemeyer, 1981). In the economic-hierarchical domain, individuals differ in their preference for intergroup dominance, social and societal hierarchy, and (ingroup) status, superiority, and power (versus egalitarianism and equality; Middendorp, 1978). This dimension is often labeled *Social Dominance Orientation* (SDO; Pratto, Sidanius, Stallworth, & Malle, 1994). The ‘conservative’ poles of both dimensions of social-ideological attitudes are related to less harmonious and more conflict-oriented views towards outgroups in particular, and more negative intergroup relations at large.

Yet, individuals do not live in an isolated societal vacuum. Instead, they are embedded in their everyday environments and communities – the neighborhoods, municipalities, states, or countries in which they live and make sense of their social world. Sociology has often argued that these contexts color individuals’ experiences with and their perceptions of outgroups. Addressing the Gordian knot of intergroup relations from a contextual differences framework, sociologists have proposed a variety of social-environmental and situational factors that may lead individuals to propagate prejudice towards outgroups (e.g., Oliver & Mendelberg, 2000).

For instance, Relative Deprivation Theory (Davis, 1959; Smith, Pettigrew, Pippin, & Bialosiewicz, 2012) holds that individuals living in dangerous, disadvantaged, and impoverished areas as opposed to safe, prosperous and affluent areas, may perceive threat in terms of their safety and welfare, which primes intergroup hostility (LeVine & Campbell, 1972). Objective environmental markers such as crime and unemployment might thus yield a negative impact on intergroup dynamics. Even the mere presence of outgroups might evoke conflict, though this claim has been fiercely contested (Hewstone, 2015; Schmid, Al Ramiah, & Hewstone, 2014; van der Meer & Tolsma, 2014).

Fifty-odd years ago, Allport (1954) concluded his chapter on the size and density of minority groups stating: “Growing density [of an ethnic-cultural minority group], therefore, is not in itself a sufficient principle to explain prejudice. What it seems to accomplish is the *aggravation* of whatever prejudice exists” (p. 229). This statement was not given much scholarly attention until political scientist Robert Putnam (2007) refuted this proposition, and posited that more ethnic diversity was associated with less trust and more prejudice between and within ethnic groups. This constrict claim, also called the “hunkering down” hypothesis, as such specified that individuals “pull in like a turtle” (Putnam 2007, p. 149), withdraw from others and from social life in the face of diversity.

But does diversity inevitably have such devastating consequences for social cohesion within communities? In a comprehensive review of 90 post-Putnam studies, van der Meer and Tolsma (2014) concluded that, at best, evidence for Putnam’s claim is mixed. Nonetheless, the academic debate on how ethnic diversity in society affects social cohesion has had significant impact on the public sphere, and still remains a hot topic to examine with no clear answers yet.

Finally, objective environmental features do not stand alone, they are inextricably intertwined with social environmental features. Indeed, the objective environment – and our perceptions thereof – as well as norms, values, and the beliefs of others surrounding us permeate all spheres of our social lives (see Guimond et al., 2013). Furthermore, such ideological contextual *climates* provide individuals with normative reference knowledge that guides their positioning towards social objects, and hence might bear an effect on our outgroup attitudes. As such, a final key issue that I address within this overview, is the question of how strongly the perception of these norms and normative climates guide individuals in their stances on various facets of intergroup relations.

## Towards a Contextual Social Psychology

The study of intergroup relations has greatly benefited from both the individual differences approach and the contextual differences approach. Yet, the role of individual and of society has often been meticulously (and artificially) separated due to field specialization. Psychology has claimed the individual as unit of analysis whereas sociology focused mainly on societal factors. An integration of individual and contextual/societal antecedents of attitudes and behavior has long been lacking. Rather, a person *versus* situation debate was dominant, in which both fields competed against each other for a clean shot at “true” variance in social phenomena. Social psychology, however, can benefit greatly from being at the intersection between psychology and sociology. Indeed, the Gordian knot of intergroup relations seems hard, if not impossible, to capture and unravel from a single standalone perspective.

Luckily, although both approaches diverge in their views on the role of the individual or the social context in shaping attitudes, they complement each other and together they can provide a useful theoretical framework for an integrated study of social-psychological phenomena. According to Doise (1986), it is a core responsibility of social psychology to bridge these levels of analysis. This focus was already articulated by Kurt Lewin’s (1936) classic heuristic equation: Behavior = *f*(Person, Environment), and also more recently, Thomas Pettigrew (1991) argued that within the arena of social psychology, examining variation across individuals and across situations should be the norm, and systematic research attention should be paid to a *Person* × *Context* approach. Importantly, he maintained that such integration should not be limited to the simultaneous consideration of individual and societal characteristics, but should expand across levels, that is, by applying a so far largely under-explored *interaction* approach (e.g., Mischel, 2004).

This overview is therefore the product of a growing scholarly interest in applying this Person × Context interplay to the field of intergroup dynamics (see also Duckitt, 2001; Hodson & Dhont, 2015; Ross & Nisbett, 2011), and summarizes various studies that aimed to integrate and reconcile both approaches in order to yield a more complete understanding of intergroup relations. Furthermore, to examine such interactions across levels of analyses, Oliver Christ and Ulrich Wagner have advocated and demonstrated the importance of multilevel analyses (Christ, Sibley & Wagner, 2012; Christ & Wagner, 2013). Multilevel research (typically using large-scale survey data; see Green, Fasel, & Sarrasin, 2010) is based on the assumption that individuals are embedded (i.e., nested) in territorial or geo-political contexts such as neighborhoods, municipalities, states, or even countries.

As such, parameters within the statistical model may vary at more than one level, and one can investigate main effects of predictors at the individual level of analysis, at the contextual level of analysis, and even *cross-level* interactions between predictors at both levels. Applied to our domain of interest, I will review recent research on intergroup attitudes as a function of individual social-ideological attitudes, contextual ethnic diversity, and the combination of both. Moreover, I will emphasize a number of studies that focused on individual social-ideological attitudes, contextual ideological ‘climates’, and their interaction in the prediction of prejudice.

## Galvanizing and Mobilizing Effects

A most interesting perspective for the interplay between individual and situational processes in intergroup dynamics was provided by Sniderman, Hagendoorn, and Prior (2004). They maintained that situational triggers may exert either a *galvanizing* or a *mobilizing* effect. In particular, galvanizing contexts have a strong effect on those already concerned about a particular issue, whereas mobilizing effects evoke strong reactions among those citizens that were originally not concerned about the problem. Theorizing about what constitutes a galvanizing vs. a mobilizing context is rather limited, and a clear conceptual definition is currently lacking in the literature. On the contrary, this line of research has been characterized by a strong empirical approach, where scholars have systematically examined various contextual factors to uncover whether they exert a galvanizing or mobilizing influence.

For example, cues of contextual social threat have been shown to elicit more prejudice especially among individuals with authoritarian predispositions (Stenner, 2005). This could be considered a galvanizing prime. On the other hand, in nations with more exclusionary policies, increased levels of opposition against immigration were found across the board, with mobilizing effects among individuals with conservative as well as egalitarian values (Schwartz, 2007). Within the broader person × context framework to intergroup attitudes and relations, numerous studies have examined whether various contextual features have a galvanizing or mobilizing influence. In the next section, I will highlight intriguing studies revealing the galvanizing potential of ethnic diversity, and the mobilizing potential of right-wing ideological contextual climates.

Across five empirical studies, Van Assche and colleagues found that ethnic diversity exerted a galvanizing influence, consistently (and sometimes even exclusively) having its strongest impact on those already concerned about it. Firstly, Van Assche, Roets, Dhont, and Van Hiel (2014) provided evidence for the claim made by Allport (1954) that a large presence of ethnic and cultural outgroups in itself is unlikely to trigger negative attitudes towards these outgroups. Indeed, they confirmed the general hypothesis of Sniderman and colleagues (2004) that ethnic diversity *galvanizes* only those already worried about it. In particular, they found that a large immigrant proportion solely accentuates the negative outgroup attitudes that already exist among certain individuals (e.g., those high in right-wing authoritarianism).

Other work found similar galvanizing effects of ethnic diversity, for individual differences in left-right self-placement (Karreth, Singh, & Stojek, 2015), dangerous worldviews (Sibley et al., 2013) and conformity values (Fasel, Green, & Sarrasin, 2013), three *moderators* closely related to the concept of authoritarianism (Duckitt, 2001). Importantly, recent studies corroborated that authoritarianism also moderates (i.e., strengthens) diversity’s effects on other intergroup attitudes. These contributions focused on related *outcomes* such as intergroup contact experiences (Brune, Asbrock, & Sibley, 2016; Van Assche, Asbrock, Dhont, and Roets, 2018), support for multiculturalism (e.g., Kauff, Asbrock, Thorner, & Wagner, 2013), feelings of threat, anxiety, and mistrust towards outgroups (Van Assche, Roets, Dhont, & Van Hiel, 2016), and political cynicism, mistrust, and populist party support (Van Assche, Dhont, Van Hiel, & Roets, 2018; Van Assche, Van Hiel, Dhont, & Roets, in press). In the latter study, it was revealed that, at least with regards to political attitudes and populist party support, ethnic diversity much more threatens peoples’ social-cultural motives of ingroup safety and security (captured by right-wing authoritarianism) than their economic-hierarchical motives of intergroup superiority and power (captured by social dominance orientation).

These findings all indicate that not everyone is equally sensitive to diversity, and diversity’s repercussions seem to depend on the levels of authoritarianism of the perceiver. Notably, other relevant moderators have been examined as well. For instance, Van Assche, Asbrock, Roets, and Kauff (2018) took into account the role of positive neighborhood norms, and showed that the galvanizing effect of diversity spills over to people’s views and behaviors within their local neighborhood life, making especially (or even exclusively) those who perceive negative local norms react to diversity with lower neighborhood satisfaction, greater perceived neighborhood disadvantage, and greater moving intentions. In other words, the negative consequences of diversity did not occur when perceived social norms were positive. This conclusion is crucial and can contribute to the ongoing public and political debate concerning diversity and social and communal life. Positive norms can form the “social glue” that holds communities together, with a range of positive outcomes for local neighborhood life (Putnam, 1993). Even further, such positive local norms maintain social cohesion in a neighborhood, even under a potential threat like high ethnic diversity.

A vital question that remains is whether all contextual influences are created equal. Numerous studies provided firm evidence for a galvanizing effect of ethnic diversity. Yet, Sniderman and colleagues (2004) also developed an understanding of *mobilizing* effects as situational triggers that evoke strong reactions among citizens that were originally not concerned about the issue. Such contexts that facilitate mobilizing effects are characterized by strong normative cues. As such, Van Assche, Roets, De keersmaecker, and Van Hiel (2017) introduced the concept of right-wing ideological climates as socially shared stances on social-cultural and economic-hierarchical dimensions of ideology within a certain societal context. Ideological climates can provide social groups, organizations, and even whole societies with a set of unifying, collectively shared norms and values that guide how individuals within these contexts think about, understand, and evaluate other social groups (Cohrs, 2012). Even further, ideological climates can have an impact beyond the values and beliefs individuals personally endorse on target variables like outgroup attitudes (see Guimond et al., 2013).

Some studies have made use of the presence of radical right-wing parties as an indicator of a conservative climate within a geo-political area. Two studies conducted in Switzerland revealed that individuals’ opposition to anti-racism laws (Sarrasin et al., 2012) and individuals’ attitudes towards Muslim women wearing a veil (Fasel et al., 2013) were predicted by the conservative climate of their municipality (indexed by referendum results), in addition to individual-level conservative values. However, the presence of particular political parties or referendum results is a proxy for right-wing ideological climates within a region. Therefore, Van Assche and colleagues (2017) suggested a bottom-up approach to ideological climates by using aggregated individual-level measures of values and beliefs to reflect *popular* views within a context.

Their large-scale examination obtained several cross-level interactions indicating that the slope of the right-wing social attitude – negative outgroup attitude relation is steeper in low right-wing contexts as compared to high right-wing contexts. Interestingly, this pattern was found for attitudes towards ethnicity-, gender-, and age-based outgroups. In other words: across several contexts, their findings indicated that right-wing climates mobilize those least likely to be prejudiced to adopt negative stances towards a variety of outgroups, even beyond their personally endorsed worldviews, values and beliefs. This result shows that norm setting as a mobilizing mechanism operates at different geo-political levels, expressed in everyday personal interactions as well as in local, regional, and even national institutions and decision-making processes. Interestingly, especially individuals low on right-wing social-ideological attitudes were mobilized, indicating the powerful impact of strong, exclusionary right-wing climates. Individuals strongly endorsing right-wing social-ideological attitudes, in turn, seemed little affected by low right-wing, inclusive climates.

This conclusion fits well with findings of other studies that, instead of tapping into prejudice, focused on the relationship between personality and political orientation. For instance, Sibley, Osborne, and Duckitt (2012) demonstrated that higher homicide and unemployment rates weakened the relationship between the personality trait Openness to Experience and political orientation. In particular, those high in openness (who are usually least likely to adopt right-wing views), tended to endorse a right-wing orientation up to the same level as close-minded individuals in areas characterized by high levels of homicide and unemployment. Interestingly, the powerful impact of this mobilizing contextual feature did not affect closed-minded people (who supported right-wing views regardless of where they lived). As such, mobilizing features can play a mitigating role both in the association between basic traits and ideological stances, as well as in the relation between these stances and outgroup attitudes.

In sum, across several empirical studies, it was revealed that a systematic person × context interaction approach is warranted to shed light on the intriguing interplay between ethnic diversity, ideological climates, and various facets of intergroup relations.

## General Discussion

Throughout this overview, I have highlighted the major contributions and implications of the findings of several contributions individually. Here, I will discuss some general implications of this research. Also, I will formulate some limitations and avenues for future research.

In general, I believe that scholars in social psychology and related fields should use their research findings on intergroup phenomena to help generate greater understanding of multicultural nature of our societies. At the same time, policy makers should welcome attempts from researchers to descend from their “ivory tower” and engage in societal debates. For example, in light of the sudden and considerable influx of refugees in Germany in 2015, over a hundred German social psychologists sent a petition to the German parliament.

In this “manifesto”, they bundled their social-psychological knowledge from several scientific studies and offered clear recommendations for a humanitarian welcoming of these immigrants and a well-organized handling of this abrupt rise in ethnic and cultural diversity. In this way, social-psychological knowledge accompanied the political debate, and Chancellor Angela Merkel proclaimed her most famous quote: “*Wir schaffen das*” [“We will make it work”].

### Three Major Lessons and Their Implications for Policy Making

Taken together, three major lessons can be inferred that may inform policy makers, agencies, and organizations aiming to reduce prejudice, ameliorate tolerance, and build more harmonious intergroup relations in their local communities. It is important to note that these three lessons do not stand alone. Rather, they interact with each other, and – ideally – policy making initiatives should take them into account simultaneously.

#### Lesson 1: Diversity Is Neither Good Nor Bad!

Firstly, it is important to note that ethnic diversity in a local environment is not by default harmful for intergroup relations, as Putnam (2007) notoriously claimed. In fact, diversity is a potential source of strength if it is recognized, valued, and properly managed. Across various studies, it was shown that the actual number of ethnic-cultural minorities in the local environment is not related to most intergroup outcomes. In general, the conclusion seems very straightforward: diversity is neither bad nor good in itself. In the same vein, perceptions of diversity seem to have little to do with outgroup stances, though they do play a role in combination with pre-existing social-ideological attitudes.

In particular, those low in right-wing social-ideological attitudes seem little affected by the proportion of minority members in their immediate living area. If anything, they react with even more general positive attitudes towards outgroups, though their levels of intergroup contact, outgroup threat, and political cynicism and trust remain unchanged. Contrariwise, for those high in right-wing social-ideological attitudes, diversity seems to galvanize and aggravate their previously held (negative) stances on intergroup relations. Indeed, in the face of diversity, those individuals are driven towards more prejudice, more outgroup negativity, more cynicism towards politics and politicians, more mistrust (in politic(ian)s, in outgroups, and in general), more populist party support, more outgroup threat feelings, more negative intergroup contact experiences. Interestingly, most individuals (and in particular high authoritarians) engage in more intergroup contact in diverse settings.

This latter fact is a hopeful and encouraging side-effect of diversity. In diverse environments (ideally with positive, inclusive norms), contact opportunities will be plenty, and right-wing individuals will be the ones most likely to engage in contact and benefit from its consequences (see Brune et al., 2016). Where contact opportunities are few, in turn, the visibility of minorities could be increased, so that the few contact experiences that do take place are generalized onto other outgroup members. This can be done by acknowledging ethnic-cultural minority members in local events, in advertisements, and in the media. Hence, there are plenty of opportunities for local policy makers to organize small and non-intrusive events where neighbors have the opportunity to get to know one another and learn to get along well. Whereas higher levels of diversity will unavoidably entail contact experiences that are negative in valence, positive intergroup contact is much more frequent (Graf, Paolini, & Rubin, 2014). Moreover, such interventions have been shown to effectively boost residents’ local bonds and satisfaction with their neighborhood (e.g., Kleinhans, 2009) and to promote tolerant intergroup norms and customs, which are central conditions for reducing exclusionary attitudes in culturally diverse communities and societies (Allport, 1954).

#### Lesson 2: Knowing You, Knowing Me… Individual Differences Matter!

Secondly, since long at the center of prejudice research, the social-ideological attitudes of social dominance orientation and particularly right-wing authoritarianism have proven to be firm and stable individual-level predictors of people’s stances towards intergroup dynamics. Indeed, individual differences in social-ideological attitudes explain variance in attitudes towards ethnic-cultural outgroups, towards other gender and age groups, towards politics and politicians, and even towards political party programs. Knowing which worldview an individual adheres to can thus guide agents towards specific interventions.

Again, a most excellent example resides in simple intergroup contact experiences (Allport, 1954; Pettigrew, 1998; Pettigrew & Tropp, 2006). Positive contact experiences reduce prejudice, their largest beneficial effects being among those high in right-wing authoritarianism and social dominance orientation (Dhont & Van Hiel, 2009; Hodson, 2011). Even further, social-ideological attitudes are to some degree malleable, and positive contact with outgroup members over time decreases levels of right-wing social-ideological attitudes (Dhont, Van Hiel, & Hewstone, 2014). As such, promoting intergroup contact experiences is beneficial in both local and personal interventions. In conclusion, individual differences in social-ideological attitudes matter, and they should be taken into account in policy making initiatives. Awareness of such individual differences is essential, not only because they determine how people react to diversity, but also because such knowledge provides useful information with regards to the potential effectiveness of certain person-centered approaches.

#### Lesson 3: Mind the Norms!

A third and final conclusion is that norms are key to intergroup relations. Whether they represent perceptions of the local norms and atmosphere within one’s living area, or socially shared stances within a certain societal context (i.e., normative climates), they exert a certain influence on our daily lives. For instance, individuals who perceive positive norms in their neighborhood tend to be more satisfied with their neighborhood, they are less inclined to perceive their neighborhood as disadvantaged and deteriorated, and they are less inclined to move away from their neighborhood – even when this neighborhood has a large population of ethnic-cultural minority members.

In the same vein, positive norms will likely install an optimal intergroup climate where residents of various ethnic and cultural backgrounds engage in positive contact experiences and are tolerant towards each other. Guimond and colleagues (2013) even showed that country-level pro-diversity policies can impact individuals’ anti-immigrant attitudes beyond their social-ideological attitudes (e.g., their personal level of social dominance orientation). The perception of local norms plays a crucial role here, once again hinting at norm setting as potential mechanism behind certain contextual effects.

On the other hand, right-wing normative climates can instill a hostile and exclusionary intergroup atmosphere, fueling tension, conflict, and feelings of threat, anxiety and mistrust towards “them” (i.e., the outgroup). Somewhat ironically, such right-wing climates seem to primarily affect those who are the least “prone to prejudice”. That is, right-wing climates particularly mobilize those low in right-wing social-ideological attitudes towards heightened outgroup negativity. This situation indicates that norms and intergroup relations are inextricably intertwined, for better and for worse.

This conclusion further demonstrates that local policy makers should be extremely wary when propagating initiatives that target local norms. No matter how noble and honorable, if their interventions don’t succeed, the backlash might be disastrous. Indeed, failed interventions might push especially the most tolerant individuals towards more hostile outgroup opinions. Hence, intergroup dynamics are delicate and fragile, and should be handled with care and caution.

### Further Avenues for Theoretical Fine-tuning

The person × context interaction perspective definitely has its merits, serving as a novel approach to the study of intergroup relations in real-world societal contexts (see Duckitt, 2001; and Ross & Nisbett, 2011 for excellent overviews). It is my personal opinion that the field can move forward in several additional ways, which were not directly addressed within this critical review. Indeed, apart from extracting practical implications, the promotion of these bundled findings into theoretical advancements could be of added value too. I will touch upon a few potentially fruitful paths for future theorizing.

#### What Makes a Context Galvanizing or Mobilizing?

A first point that needs to be addressed is the necessity for more elaborated research on what defines and what is measured by a galvanizing versus mobilizing context. A clear demarcation of galvanizing and mobilizing effects is lacking in the current state of the literature. As such, this vital question is open to philosophical deliberations. In my personal view, a key factor in galvanizing contexts is that they are ambiguous. Ambiguous contexts that do not provide clear normative cues for individuals (such as the mere presence of a large immigrant proportion) may galvanize the bigoted views in right-wing individuals, while they might stimulate tolerance in more broad-minded individuals.

In other words, galvanizing contexts lack clear normative guidance, are not a priori beneficial or detrimental for intergroup dynamics, but rather provide opportunities to express one’s values and act upon one’s beliefs (see Katz, 1960). Indeed, individuals not only react to and make sense of their surroundings (cf., authoritarians’ heightened feelings of threat, anxiety and mistrust in the face of diversity), they also actively shape this environment (cf., authoritarians engaging in more positive and more negative intergroup contact in diverse settings). At first, diversity as a galvanizing context could thus enlarge the prejudice-gap between those low and those high in right-wing social-ideological attitudes. In the long run, however, contact quantity (which strongly correlates with positive contact quality; see Barlow et al., 2012) should make those high in right-wing social-ideological attitudes more tolerant, up to similar levels as those low in right-wing social-ideological attitudes. Future social-psychological research could tap into the alleged ambiguous nature of (ethnic) diversity, as such shedding light on why exactly it exerts a galvanizing effect on almost every aspect of our daily intergroup reflections.

A second imperative enquiry is what makes a context or stimulus mobilizing, and why are right-wing ideological climates mobilizing? I believe that contexts facilitating mobilizing effects are characterized by strong normative cues, which especially amplify prejudices within those low in right-wing social-ideological attitudes. Specifically, there is a basic, universal human need for safety and security (e.g., Chen, Van Assche, Vansteenkiste, Soenens, & Beyers, 2015). Oesterreich (2005) has theorized that under relatively safe circumstances, the ‘natural’ variation in individual differences is not constrained, providing a balance on the left-right continuum and, accordingly, on the tolerance-prejudice continuum. However, under strong, normative circumstances (e.g., high homicide and unemployment rates; see Sibley et al., 2012), he claimed that experiences of unsafety in all citizens may disturb this balance, causing in particular those low in “authoritarian tendencies” to respond with the “rejection of the new and the unfamiliar, a rigid adherence to norms and value systems, an anxious and inflexible response to new situations, hostility, and aggression” (Oesterreich, 2005, p. 275).

In the same vein, I argue that a right-wing ideological climate in terms of a far-right local government (e.g., Fasel et al., 2013) or a higher-level, shared level of right-wing views (Van Assche et al., 2017) imposes strong norms that entail the acceptance of and support for traditional rules, social order and social inequality. Such powerful norm setting mechanism has its greatest impact on those originally not inclined to support such beliefs (i.e., those low in right-wing attitudes). Yet again, the norm setting power of such mobilizing effects could drop over time. Examinations of prejudice in Western societies over the past decades have taught us that what used to be consensually accepted in the past (e.g., blatant racism) is now (almost) consensually rejected (see Pettigrew & Meertens, 1995).

In the long run, processes such as collective action might thus counter the adoption of anti-outgroup stances among broad-minded individuals in right-wing climates. In that respect, future studies could purposefully select individuals across different contextual levels (ranging from peer groups to neighborhoods, regions, and countries) in which they assess both ethnic diversity, individual social-ideological attitudes and contextual ideological climates within each level. Various person × context and context × context interactions between these constructs could then be tested in order to provide a more elaborated view on the interplay between various intergroup mechanisms at different levels of analysis.

#### The Bigger Picture: Connecting the Dots with Other Fields

Apart from fine-tuning our theorizing of galvanizing and mobilizing effects, integrating the findings of this stream of research within other segments of the social-psychological literature might be a fascinating avenue for future research. For instance, the minority perspective remains relatively underexposed. This is a pity, since it might yield a new and potentially diverging viewpoint on the intergroup dynamics literature. John Berry (1984) offered a framework to help understand the different types of identity processes that immigrant groups experience within the dominant culture of the host society. Dovidio, Gaertner, and Kafati (2000) later adapted this acculturation framework to represent intergroup relations between majority and minority groups more in depth.

Within this body of literature, Van Zomeren, Postmes, and Spears (2008) expanded the premise that effects found for majority members don’t necessarily translate into similar effects for minorities. In short, they maintained that for minority members, intergroup contact experiences with majority members would negatively predict collective action and support for policies benefiting the ingroup (i.e., the “sedative” effect of intergroup contact). This finding became topic of heated scholarly debates (e.g., Cakal, Hewstone, Schwär, & Heath, 2011), but – to my knowledge – was never united with the knowledge on ethnic diversity effects and the effects of (both individual and contextual) norms.

Similarly, sociologist Eric Uslaner (2012) proposed that it is segregation rather than diversity that is detrimental to social cohesion. Intergroup contact plays a significant role in prejudice reduction at various societal levels, but its potential to improve intergroup relations and shape tolerant norms cannot be fully expanded when there are no opportunities. Luckily, indirect contact experiences also impact a large number of people who do not themselves experience such contact (Wright, Aron, McLaughlin-Volpe, & Ropp, 1997). Mere knowledge of ingroup members having positive interactions with outgroup members can boost one’s tolerance levels. Even individuals living in a segregated neighborhood within a diverse city can benefit from living in such mixed contextual setting where fellow ingroup members do engage in intergroup contact, even if they themselves rarely experience such direct, face-to-face intergroup encounters (see Christ et al., 2014).

#### The WEIRD World is Not Enough

As a final avenue for future research, I would like to highlight the need for cross-cultural validations of our results. As an overwhelming majority (i.e., 96%) of samples for psychological studies are drawn from Western, Educated, Industrialized, Rich, and Democratic (“WEIRD”) societies, research across cultures has been put high on the research agenda, to allow for generalizations of research findings (Henrich, Heine, & Norenzayan, 2010). In Europe, there is ample evidence on how intergroup tensions arise due to immigration (see Green et al., 2010; Hewstone, 2015). In other countries, however, intergroup dynamics developed in a totally different historical setting. Hence, generalizations of our results are not self-evident, as portrayed in the example below.

Roets, Au, and Van Hiel (2015) considered the history of ethnic conflict (between Chinese, Malays, Indians, and Eurasians) in Singapore, which has been tense, and even nowadays, the encouragement of positive changes at social and societal levels is a hot topic in political discussions. The provocative findings of this particular study challenged the widely adopted postulate that authoritarianism is inevitably associated with increased prejudice. In particular, their results demonstrated that in Singapore, a positive relation was found between right-wing authoritarianism and positive outgroup attitudes. The rationale is that the government as a strong authority explicitly and relentlessly endorses diversity and multiculturalism, thereby enforcing a social norm that is in direct opposition to authoritarians’ “natural” negative stances towards other social groups. This illustrative case shows that wherever several groups live together, studies that provide a better understanding of possible pathways towards positive intergroup relations are highly relevant (cf. Van Assche, Bostyn, De keersmaecker, Dardenne, & Hansenne, 2017).

## Conclusion

In the present overview, I started from the rather negative and pessimistic view of Putnam (2007) on ethnic diversity that has dominated the literature on intergroup relations. My aim was to investigate this complex issue from a person × context interaction perspective, elaborating on the more nuanced hypothesis of Allport (1954) that diversity only aggravates the already-present prejudice in certain individuals. Across various studies, I consistently found evidence that supports this nuanced interpretation. Furthermore, I reviewed several studies aiming to increase our understanding of how ideological climates as shared, collective norms guide how certain individuals feel, think about, and behave towards other social groups. Where diversity showed the potential to magnify the influence of individual differences (i.e., as a galvanizing context), right-wing ideological climates attenuated them by steering everybody into a certain direction (i.e., as a mobilizing context).

As such, the present review offers important new insights in the complex and fascinating research domain of ethnic diversity, ideological climates, and intergroup dynamics in particular. As Pettigrew (2017) pointed out: “New theory and methods have aided social psychology to begin to situate its phenomena in their broad social contexts. This is an extremely significant advance in the discipline that should be celebrated and continued. Contextual social psychology is finally emerging.”
